# Prevalence of radiographic misinterpretation of urethral bulking agents: a retrospective, single-center study

**DOI:** 10.1007/s00261-025-05326-8

**Published:** 2025-12-19

**Authors:** Rose Darcy, Margot Barker, John Masterson, Rachel Caskey, Clarissa Niino, Karyn Eilber

**Affiliations:** 1 Feinberg School of Medicine, Northwestern University, Chicago, USA; 2 Department of Obstetrics and Gynecology, Cedars-Sinai Medical Center, Los Angeles, USA; 3 Department of Urology, Cedars-Sinai Medical Center, Los Angeles, USA

**Keywords:** Urethral bulking agents, Misinterpretation, Periurethral, Bladder stone, Imaging, Stress urinary incontinence

## Abstract

**Purpose:**

Urethral bulking agents (UBA) are a treatment option for patients with stress urinary incontinence and can contain radiopaque material such that misdiagnosis for pelvic pathology occurs. We aim to characterize radiologic interpretation of UBA in patients who had computed tomography of the abdomen and pelvis (CTAP) after UBA.

**Methods:**

All patients who underwent UBA from January 2011 to March 2024 were identified in the electronic medical record (EMR). Charts were reviewed to identify patients who underwent subsequent CTAP. Radiologic interpretations were reviewed to assess for accuracy in identification of the UBA as well as patient communications regarding imaging results.

**Results:**

During the study period, 144 patients underwent injection of UBA. Of those, 31 patients had ≥ 1 CTAP for a total of 61 CTAPs. Average duration of UBA to CTAP was 25.7 months. The UBA was accurately identified in only 15 (24.6%) scans. In 31 (50.8%) scans, neither UBA nor any abnormality was noted, and one of the authors confirmed presence of UBA in 30 of the 31 scans. UBA was inaccurately characterized as bladder stones (*n* = 7), nonspecific calcifications (*n* = 6), radiodense material (*n* = 1), and a urethral stone (*n* = 1) in the remaining 15 (24.6%) scans. Two patients with UBA who were misdiagnosed as bladder stones messaged via the patient portal about the findings with one expressing anxiety regarding the finding.

**Conclusions:**

Urethral bulking agents are often inaccurately interpreted on CTAP potentially leading to anxiety and unnecessary intervention. This highlights the importance of raising awareness of UBA among radiologists.

## Introduction

Stress urinary incontinence (SUI) is the most common cause of urinary incontinence and is one of the most prevalent health conditions for women worldwide, estimated to affect more than 40% of females [1, 2]. Although SUI is more common in women, SUI also impacts men, with one in three men developing SUI after prostatectomy.

Conservative management is first-line for treatment of SUI and includes lifestyle interventions such as weight loss, pelvic floor physical therapy, and/or pessary placement to support the urethra [1]. Surgical intervention is the most effective treatment with mesh urethral slings considered the gold standard for treatment of SUI.

Urethral bulking agents (UBA) are an effective alternative treatment for patients that fail conservative therapy but desire a less-invasive intervention than surgery. Bulking agents have been available for nearly a century and primarily fall into two classes: particulate bulking agents and homogenous gel-type bulking agents. Both particulate and homogenous gel-type bulking agents function by causing reactivity in local tissues that lead to stable, permanent integration of the bulking agent into the urethral submucosa, thereby increasing urethral resistance at rest and preventing incontinence with increases in abdominal pressure [1, 3].

Bulking agents can be both radiopaque and space occupying. As such, they may be visualized by a variety of imaging modalities. However, they are often not indicated as part of the patient history provided when ordering abdominal or pelvic imaging. [2] Without this pertinent patient history, urethral bulking agents are often incidentally found and misdiagnosed on subsequently performed imaging studies, which can lead to significant anxiety and/or unnecessary intervention [2, 4]. The objective of the present study was to characterize accuracy of radiologic interpretation of a calcium hydroxylapatite urethral bulking agent (Coaptite^®^) in patients who underwent subsequent imaging with computed-tomography.

## Materials and methods

This study was deemed exempt by the Cedars Sinai Institutional Review Board. All patients who underwent injection of a urethral bulking agent for stress urinary incontinence at our institution from January 2011 to March 2024 were identified in the electronic medical record (EMR) using Current Procedural Terminology (CPT) code 51715 (transurethral endoscopic injection of implant material into the submucosal tissues of the urethra and/or bladder neck). The urethral bulking agent utilized at our institution during the study period was comprised of calcium hydroxylapatite (Coaptite^®^). All charts were reviewed to identify the subset of patients who underwent computed tomography of the abdomen and pelvis (CTAP) after injection of a urethral bulking agent. Patients were included in our analysis if they had undergone urethral bulking agent injection and had subsequent CTAP for any indication. Exclusion criteria included patients without subsequent CTAP or without available radiology report from their abdominopelvic imaging. Additionally, because previous radiological reports were available to the radiologists reporting subsequent CTAPs, scans completed and read after the urethral bulking agent had been correctly identified in a previous CTAP were excluded. Radiology reports of all studies performed for the subset of patients were reviewed by one author (initials JM) to assess for accuracy in determination of the presence of a urethral bulking agent, and actual images were individually reviewed (JM) for correlation with the radiology report when necessary to confirm diagnoses. The primary outcome was misinterpretation of urethral bulking agent in subsequent abdominopelvic imaging reports. Secondary outcomes included patient communications regarding imaging results recorded in the EMR, which were individually reviewed by study authors.

## Results

One hundred forty-four patients underwent urethral bulking agent injection over the course of the study period (January 2011 to March 2024). Of those 144 patients, 31(21.5%) went on to have subsequent abdominal imaging with one or more CTAP, for a total of 61 CTAPs. On average, patients underwent CTAP 25.7 months (range 1.1 to 85.8 months) after injection of urethral bulking agent. The analyzed CTAP were performed and reported by multiple institutions. None of the patients underwent diagnostic cystoscopy under the care of the senior author. The urethral bulking agent was accurately identified in 15 scans (24.6%). In just over half of the scans done after injection of urethral bulking agent (*n* = 31, 50.8%), the resultant imaging report failed to describe the bulking agent at all despite the urethral bulking agent being identifiable in all but one scan when reviewed by one study author (JM). The remaining 15 (24.6%) of radiology reports identified but inaccurately characterized urethral bulking agent. The mischaracterizations of bulking agents included: bladder stones (*n* = 7), nonspecific calcifications (*n* = 6,) radiodense material (*n* = 1), and urethral stone (*n* = 1), shown in Table 1.

**Table 1:** Overall population CTAP interpretation

CTAP Findings After Urethral Bulking	*n*	%
Incorrectly identified	45	73.8%
Not reported	30	
Bladder stone	7	
Nonspecific calcifications	6	
Radiodense material	1	
Urethral stone	1	
Correctly identified	15	24.6%
Absent on scan	1	1.6%

**Fig. 1:**
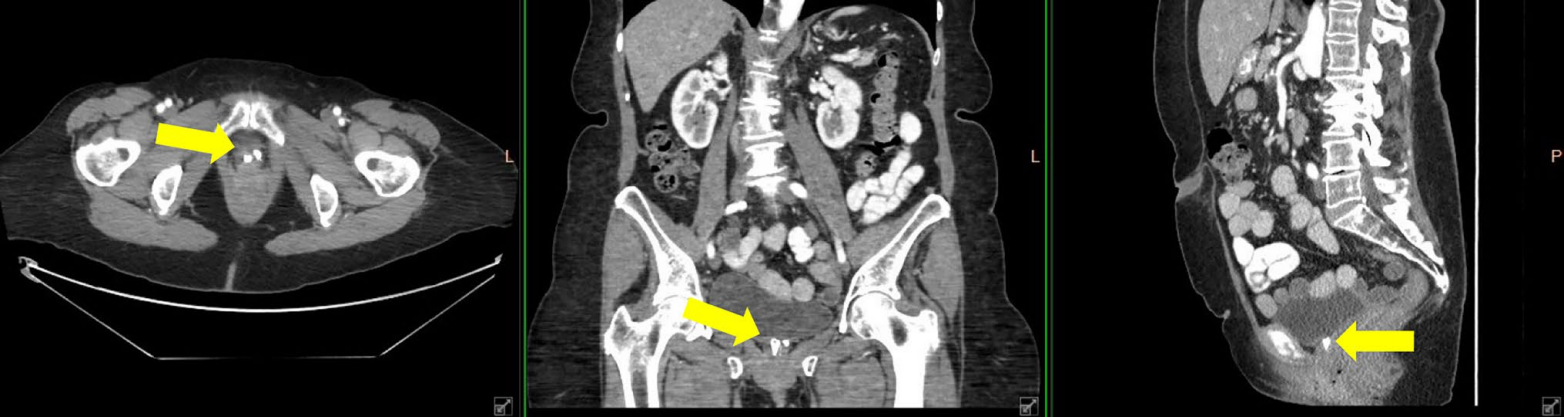
CTAP image of urethral bulking agent interpreted as “bladder stone

Manual chart review of the electronic medical records for all patients with mischaracterized urethral bulking agent on CTAP done after injection revealed that two patients (25%) reached out to their healthcare team to discuss the imaging findings. In both of these patients, the bulking agent was falsely characterized as a bladder stone, shown in Fig. 1. One of the patients expressed anxiety about the findings and sought treatment recommendations for management of the condition.

## Discussion

Urethral bulking agents are a minimally invasive treatment of stress urinary incontinence; they are thought to work by causing reactivity in local tissues leading to permanent integration of the bulking agent into the urethral submucosa, thereby increasing urethral resistance. By nature, they are space-occupying and can often be visualized on a variety of imaging modalities. However, our study determined that bulking agents are incorrectly characterized more than 75% of the time. Furthermore, the presence of a bulking agent was either not mentioned despite being identifiable upon imaging or incorrectly identified as pathologies such as bladder stones, nonspecific calcifications, radiodense material, or urethral stones. This mischaracterization can lead to patient anxiety and/or unnecessary evaluation and intervention, particularly in light of legislation that gives patients early access to their medical records.

A similar phenomenon was noted with use of dextranomer/hyaluronic acid copolymer (Deflux) for treatment of vesicoureteral reflux in children in a study by Cerwinka *et* al. which described the bright appearance of Deflux implants on T2-weighted MRI imaging done after the procedure [5]. However, to our knowledge, there are few publications related to this phenomenon in urethral bulking agents: a pictorial essay published by Bridges et al. in 2004, a study by Gaines et al. in 2018, and two case reports highlighting difficulty differentiating urethral bulking agent from periurethral diverticula and PET-avid lesions, respectively [2, 3, 6, 7]. The pictorial essay aimed to characterize the radiographic, CT, and MRI appearance of some urethral bulking agents due to their expanding clinical use as treatment for stress urinary incontinence and the assumption that radiologists would be expected to recognize their imaging features in multiple modalities in order to avoid diagnostic pitfalls [3]. Their description of bulking agents on CT included the following: bovine collagen agents have variable appearance on CT as collagen is avascular and the appearance depends on the density of surrounding tissues; carbon-coated microbeads (old-formulation of Durasphere^®^) appear at least as dense as cortical bone on CT; graphite coated microbeads (new-formulation of Durasphere^®^) appear less-dense on CT and mimic the appearance of medullary bone [3]. This study does not detail the appearance of newer bulking agent formulations such as Macroplastique^®^ and Urolastic^®^ (silicone), Coaptite^®^ (calcium hydroxylapatite), or Bulkamid^®^ (polyacrylamide). Detailed characterization of the appearance of these bulking agents on various imaging modalities is an important opportunity for future investigation.

Years later, Gaines et al. reported high rates of mischaracterization of bulking agents on subsequent abdominopelvic imaging, which they attributed to omission of the bulking agent from the patient-specific medical history provided to radiologists in the imaging order [2]. This context is critical to appropriate diagnosis and characterization of bulking agents, as they otherwise appear as vague space-occupying or calcified material. It was theorized that this dilemma would resolve with the near-universal adoption of electronic medical record systems which would create a centralized route for reporting patient medical and surgical histories to radiologists when ordering imaging. However, our study demonstrated that this was not the case, as urethral bulking agents continue to go unnoted or mischaracterized.

The profound paucity in literature discussing the frequent mischaracterization of urethral bulking agents may contribute to the persistence of this trend [2, 3]. In order to avoid the pitfall of mischaracterizing urethral bulking agents, it will be important to raise awareness of urethral bulking agents among radiologists both to increase the accuracy of diagnosing the presence of bulking agents and mitigate the anxiety and potentially unnecessary testing for patients. In addition, it is the responsibility of physicians who perform injection of urethral bulking agents to inform patients that their bulking agent will be visible on imaging when appropriate. Length of time between treatment and subsequent imaging may have contributed to the mischaracterization of bulking agents; as based on patient communication following imaging, many of the patients simply forgot they had a urethral bulking agent. Furthermore, following injection of a urethral bulking agent, clinicians need to ensure that patients understand that the bulking agent may be present and identifiable on imaging indefinitely.

## Conclusions

Urethral bulking agents are frequently mischaracterized on subsequent abdominopelvic imaging done after injection; bulking agents either went unmentioned despite being identifiable upon imaging or were incorrectly identified as pathologies such as bladder stones, nonspecific calcifications, radiodense material, or urethral stones. This mischaracterization can cause patient anxiety and lead to unnecessary intervention. It is important to raise awareness of urethral bulking agents and their appearance on abdominopelvic imaging amongst radiologists and to counsel patients clearly on the possibility that their bulking agent will be visible on subsequent imaging studies so that they may advocate for themselves. Ideally in the future, possibly with the aid of artificial intelligence, an automatic notice would be sent to the radiologist when a CTAP has been performed for someone with a history of urethral bulking agent injection. Demographic characteristics and the clinical setting in which the imaging was ordered (elective, urgent, STAT, etc.) were out of the scope of this study, however, future research should be aimed at investigating the effects of these variables on rates of misinterpretation of bulking agents.

## Limitations

Strengths of this study include the relatively large cohort and real-world imaging interpretation across multiple institutions. The results of the present study are limited by the fact that it includes only one bulking agent. Additional research is necessary to characterize appearance of other available bulking agents on imaging. However, as bulking agents are generally radiodense and space-occupying by nature, we anticipate that the conclusions and implications of the present study remain applicable. Additionally, outcome correlation (visibility of bulking agents on CTAP vs. continence/symptom burden) was not possible due to unavailable clinical data; this may be explored in future work.

## Data Availability

The data that support the findings of this study are not openly available due to reasons of patient privacy and are available from the corresponding author upon reasonable request.

## References

[1] Sikora M et al (2024) *Current Treatment of Stress Urinary Incontinence by Bulking Agents and Laser Therapy-An Update*. J Clin Med. 10.3390/jcm13051377 38592248 10.3390/jcm13051377 PMC10932143

[2] Gaines, N., et al., *Radiographic Misdiagnoses After Periurethral Bulking Agents*. Female Pelvic Med Reconstr Surg, 2018. 24(4): p. 312–314. 28657999 10.1097/SPV.0000000000000440

[3] Bridges, M.D., S.P. Petrou, and D.J. Lightner, *Urethral Bulking Agents: Imaging Review*. American Journal of Roentgenology, 2005. 185(1): p. 257–264. 15972433 10.2214/ajr.185.1.01850257

[4] Mezrich, J.L., et al., *Patient Electronic Access to Final Radiology Reports: What Is the Current Standard of Practice, and Is an Embargo Period Appropriate?* Radiology, 2021. 300(1): p. 187–189. 33944630 10.1148/radiol.2021204382

[5] Cerwinka, W.H., et al., *Appearance of Deflux implants with magnetic resonance imaging after endoscopic treatment of vesicoureteral reflux in children*. J Pediatr Urol, 2009. 5(2): p. 114–8. 19019734 10.1016/j.jpurol.2008.10.005

[6] Herforth C, Zimmern PE (2024) Macroplastique^®^ for stress urinary incontinence lights up as a PET-avid urethral lesion: A case report. Case Rep Womens Health 43:e00649 39314984 10.1016/j.crwh.2024.e00649 PMC11418125

[7] Akinjise-Ferdinand, O., et al., *A diagnostic conundrum: Is it a periurethral diverticulum/cyst or a bulking agent (Bulkamid)*? Neurourol Urodyn, 2023. 42(2): p. 547–554. 36285552 10.1002/nau.25068

